# Supplementary material for: Oxygen isotope (δ18O, Δ′17O) insights into continental mantle evolution since the Archean
Source: Nat Commun. 2022 Jul 4;13:3779. doi: 10.1038/s41467-022-31586-9 (PMC9253152; doi:10.1038/s41467-022-31586-9)
Supplement: Supplementary file 1 — Supplementary Information [file 41467_2022_31586_MOESM1_ESM.pdf]

SUPPLEMENTARY FIGURES 1-3 and Supplementary references for studied samples

Effects of Different Age assignment on trends

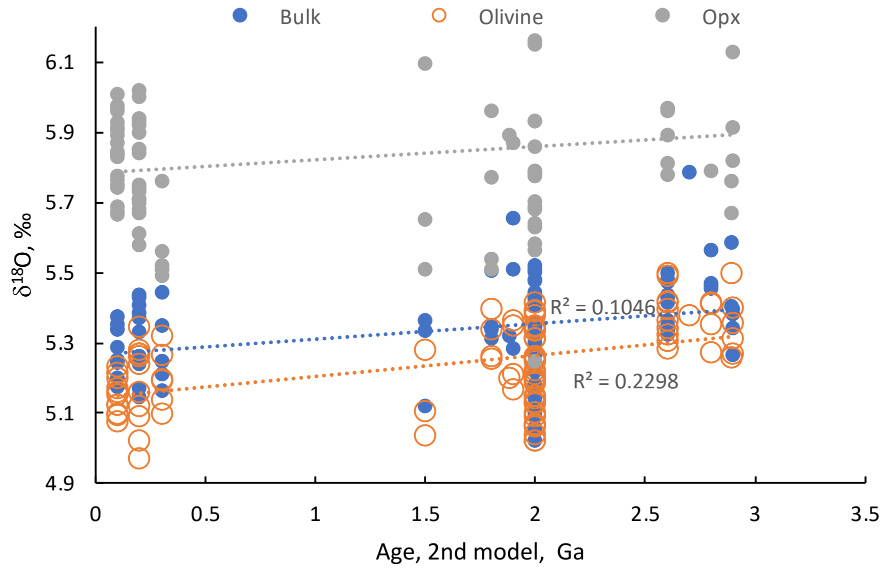

Supplementary Fig. 1. Data from this study plotted vs different age assignments (Age 2, Supplementary Table 1) based on the age of the lithosphere and the youngest possible age assignment assuming the latest rejuvenation. Compare to Fig. 1 in the text, younger age assignment does not change the decreasing trend.

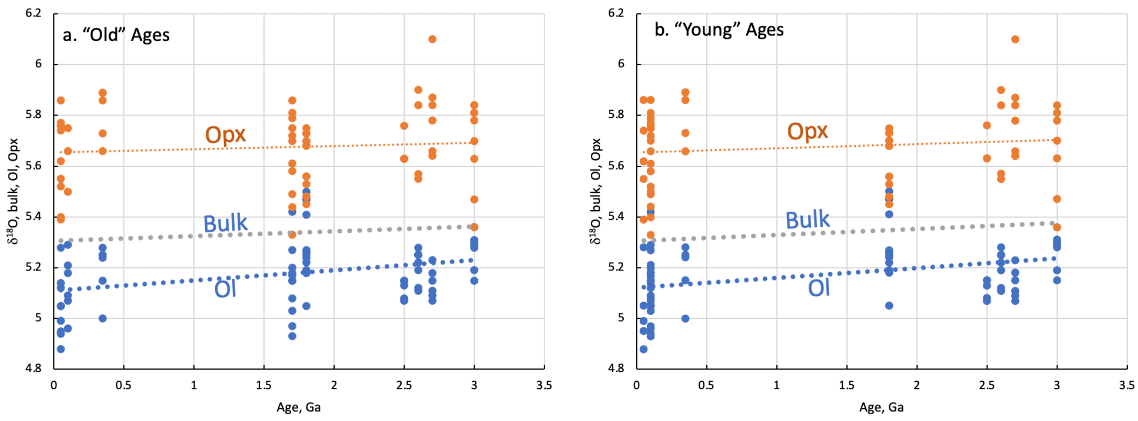

Supplementary Fig. 2. Data from Mattey et al. (1994) plotted vs continental lithospheric age. Notice decreasing $\delta^{18}O$ trends similar to what we found in this paper. Notice that both $\delta^{18}O_{Ol}$ and $\delta^{18}O_{Bulk}$ are decreasing with age while $\delta^{18}O_{Opx}$ stays constant or decreases, while $\Delta^{18}O_{Opx-Ol}$ stay nearly constant. Panel a) assumes the age of the mantle nodule as the age of the lithosphere they are collected from using Sm-Nd model ages (e.g. Bennett and DePaolo, 1987), while panel b) assumes that some of them represent the age of the crust modification by subduction/orogenesis, for example, S Dish Hills (California) is plotted at 0.05 Ma (Luffi et al. 2009).

Computation of average xenolith $\delta^{18}O$ values

We used measured $\delta^{18}O$ values of coexisting minerals, their measured modal abundance for most nodules, and fractionation factors from [Chiba et al. 1989 and Rosenbaum et al. 1994] at measured peridotite storage temperatures, all parameters are listed in the Supplementary Table 1. Where no pyroxene $\delta^{18}O$ data were available, we relied on computed $\delta^{18}O_{Px}$ values based on $\Delta^{18}O_{Olivine\text{-}pyroxene}$ fractionation factors to compute the expected $\delta^{18}O$ values of phases other than olivine, e.g. $\delta^{18}O_{Cpx} = \delta^{18}O_{Ol} - \Delta^{18}O_{Olivine\text{-}Clinopyroxene}$. Such procedure is robust and introduces no bias, Supplementary Figure 3 compares measured vs computed (based on $\delta^{18}O_{Olivine}$ only) bulk xenolith $\delta^{18}O$ values. In several cases when no modal mineral abundance was available, we used average modal abundance for harzburgite. Given that olivine is a predominant phase in mantle peridotites and in studied samples (70-80%), small (0.4-0.7‰) $\Delta^{18}O_{Olivine\text{-}pyroxene}$ value at mantle temperatures, and small modal differences in mineral content, such procedures result in little error. Further correction from bulk $\delta^{18}O_{Bulk}$ to $\delta^{18}O_{Original\ Peridotite}$ due to estimated melt depletion is explained in Fig. 3.

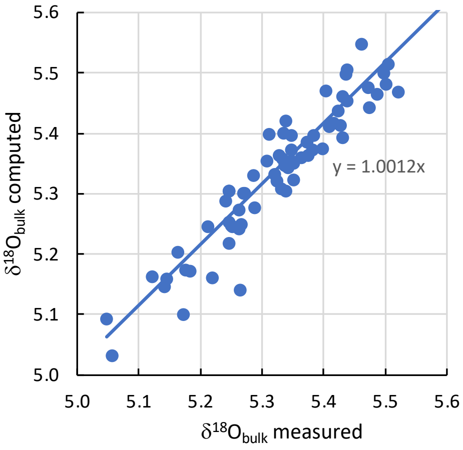

Supplementary Fig. 3 Estimation of the bulk $\delta^{18}O$ value based on measured $\delta^{18}O$ values of Olivine and Opx (± Cpx, where available) at indicated temperatures in Table A1 vs bulk $\delta^{18}O$ value computed only on $\delta^{18}O$ values of Olivine (computed). Both calculated and measured values use measured modal proportions of minerals in each peridotite. Note 1:1 agreement. This graph indicates that computing bulk peridotite $\delta^{18}O$ value based on only olivine (>75% of rock volume) and mineral proportions is robust.

References for the Supplementary Material

Mattey, D., Lowry, D. & Macpherson, C. Oxygen isotope composition of mantle peridotite. *Earth Planet. Sci. Lett.* **128**, 231–241 (1994).

Bennett, V.C. & D. J. DePaolo Proterozoic crustal history of the western United States as determined by neodymium isotope mapping. *Bull. Geol. Soc. America* **99**, 674-685 (1987).

Luffi, P., J. B. Saleeby, C.-T. A. Lee, and M. N. Ducea (2009), Lithospheric mantle duplex beneath the central Mojave Desert revealed by xenoliths from Dish Hill, California, *J. Geophys. Res.*, **114**, B03202, (2009).

Chiba, H., Chacko, T., Clayton, R.N., Goldsmith, J.R. Oxygen isotope fractionations involving diopside, forsterite, magnetite, and calcite: Application to geothermometry. *Geochim Cosmochim Acta* **53**, 2985–2995 (1989)

Rosenbaum, J.M., Kyser, T.K., Walker, D. High-temperature oxygen isotope fractionation in the enstatite-olivine-BaCO3 system. *Geochim. Cosmochim. Acta* **58**, (2653-2660 (1994)

Background references information on samples in Supplementary Tables 1-3

Doucet, L.S., Ionov, D.A., Golovin, A.V., Pokhilenko, N.P. Depth, degrees and tectonic settings of mantle melting during craton formation: inferences from major and trace element compositions of spinel harzburgite xenoliths from the Udachnaya kimberlite, central Siberia. *Earth Planet. Sci. Lett.* **359-360**, 206-218 (2012) doi: 10.1016/j.epsl.2012.10.001.

Doucet, L.S., Ionov, D.A., Golovin, A.V. The origin of coarse garnet peridotites in cratonic lithosphere: new data on xenoliths from the Udachnaya kimberlite, central Siberia. *Contrib. Miner. Petrol.* **165**, 1225-1242 (2013), doi:10.1007/s00410-013-0855-8.

Ionov, D.A., Guo, P, Nelson, W.R., Shirey, S.B., Willbold, M. Paleoproterozoic melt-depleted lithospheric mantle in the Khanka block, far eastern Russia: inferences for mobile belts bordering the North China and Siberian cratons. *Geochim. Cosmochim. Acta* **270**, 95-111 (2020); doi: 10.1016/j.gca.2019.11.019

Ionov, D.A., Harmon, R.S., France-Lanord, C., Greenwood, P.B., Ashchepkov, I.V. Oxygen isotope composition of garnet and spinel peridotites in the continental mantle: evidence from the Vitim xenolith suite, suite, southern Siberia. *Geochim Cosmochim Acta* **58**, 1463-1470 (1994)

Ionov, D.A. and Hofmann, A.W. Depth of formation of subcontinental off-craton peridotites. *Earth Planet. Sci. Lett.* **261**, 620-634. (2007) DOI: 10.1016/j.epsl.2007.07.036

Ionov, D.A., Doucet, L.S., Ashchepkov, I.V. Composition of the lithospheric mantle in the Siberian craton: New constraints from fresh peridotites in the Udachnaya-East kimberlite. *J. Petrology* **51**, 2177-2210. (2010) doi: 10.1093/petrology/egq05.

Ionov, D.A., Doucet, L.S., Xu, Y., Golovin, A.V., Oleinikov, O.B. Reworking of Archean mantle in the NE Siberian craton by carbonatite and silicate melt metasomatism: evidence from a carbonate-bearing, dunite-to-websterite xenolith suite from the Obnazhennaya kimberlite. *Geochim. Cosmochim. Acta* **224**, 132-153 (2018) doi:10.1016/j.gca.2017.12.028

Kopylova, M.G., Russell, J.K., Cookenboo, H. Petrology of Peridotite and Pyroxenite Xenoliths from the Jericho Kimberlite: Implications for the Thermal State of the Mantle beneath the Slave Craton, Northern Canada. *Journal of Petrology* **79**–104 (1999)

Mattey, D., Lowry D., Macpherson C. G., and Chazot G. Oxygen isotope composition of mantle minerals by laser fluorination analysis: homogeneity in peridotites, heterogeneity in eclogites. *Mineral. Mag.* **58A**, 573-574 (1994)

MacGregor, I.D., Manton, W.I. Roberts Victor eclogites: ancient oceanic crust. *J Geophys Res* **91**, 14063-14079 (1986)

Tollan, PME, O'Neill HSC, Hermann J. The role of trace elements in controlling H incorporation in San Carlos olivine. *Contrib Mineralogy Petrol* **17**3, 1-23 (2018)

Tollan, PME (2014) A Tale of Two Arcs: Petrogenesis of Ultramafic Xenoliths Sampling the Upper Mantle Wedge Beneath the West Bismarck Island Arc. PhD Thesis, Durham University, http://etheses.dur.ac.uk/10758/

Supplementary Table 1 Oxygen isotopic values, ages, compositional parameters and modes of mineral abundances in 104 studied mantle peridotites for dataset averages see the bottom of the table.

Sample	Rock name	Data source	$\delta^{18}O$, ‰ VSMOW							composition, WR			T°C	Age, Ga (T-RD)	Modal Abundance, %				% Melt depletion	$\delta^{18}O$, ‰ melting degree	Age, Ga (Model 2)	Av Olivine by locality	Bulk Peridotite	Peridotite corrected for melt removal
			Ol	Ol, Av	±2se	Opx	Cpx	Grt/ Sp	Bulk	Al_2O_3	CaO	Mg#WR	BK-1990	Ga	Ol	Opx	Cpx	Sp/Gar	(est)	corrected	Ga			
Jericho: Slave Craton, Canada (PPR		Kopylova et al. (1999,2000)																						
LGS26 -M11	Gr-Sp Hzb	-"-	5.290	5.282	0.017	5.65		4.98 G/S	5.33	2.26	1.90	0.918	944	2.1	69.0	23.0	4.5	3.5	35	5.46	2.6	5.224	5.376	5.502
			5.273																					
LGS22-M1	Gr-Sp Hzb	-"-	5.122	5.106	0.032	6.10			5.36	2.26	1.90	0.918	690	2.1	69.0	23.0	4.5	3.5	35	5.49	2.6			
			5.090																					
LGS22-M5	Gr-Sp Hzb	-"-	5.301	5.283	0.113	5.89	5.76	5.32	5.43		1.90	0.918	951	2.1	69.0	23.0	4.5	3.5	35	5.56	2.6			
			5.162																					
			5.430																					
Jagersfontein: Kaapvaal Craton, S.Africa (AR)			5.240																					
JF15-09	Sp Hzb, op-rich	Unpublished	5.258	5.258		5.67			5.40	0.50	0.25	0.931	668	3	61.0	37.2		0.7	40	5.55	2.89	5.352	5.558	5.693
JF15-11	Sp Hzb, cp	-"-	5.550	5.500	0.100	5.76			5.59					3					40	5.73	2.89			
			5.450																					
JF15-15	Gr-Sp Hzb	-"-	5.377	5.377					5.79	1.05	0.45	0.932	656	3	60.0	38.7			35	5.91	2.7			
JF15-63	Gr Hzb	-"-	5.178	5.273	0.096				5.45	1.13	0.37	0.935		3	74.8	21.6		3.5	35	5.58	2.8			
			5.320																					
			5.320																					
Obnazhennaya: NE Siberian Craton, Russia (AR)																								
Obn22-13	Sp Lh	Ionov et al. (2015b)	5.470	5.415	0.110		5.49		5.47	1.82	2.20	0.918	1027	2.9	76.1	15.8	1.9	2.6	35	5.60	2.8	5.392	5.498	5.624
			5.360																					
Obn1017	Gr-Sp Hzb	-"-	5.360	5.351	0.018				5.56	1.10	2.50	0.917	706	2.9	74.8	13.0	11.2	1.0	35	5.69	2.8			
			5.342																					
Obn37-13	Sp Wehrlite	-"-	5.402	5.410	0.016	5.79			5.46	0.57	2.48	0.918	685	2.9	85.2	3.9	10.5	0.4	35	5.59	2.8			
			5.418																					
Obnazhennaya: NE Siberian Craton, Russia (PR)																								
Obn01-13	Sp Lh, opx-rich	Ionov et al. (2015b)	5.349	5.349		5.52			5.65	1.68	1.40	0.917	772	1.9	58.3	33.7	4.0	1.2	30	5.76	1.9	5.293	5.482	5.596
Obn08-13	Hzb Gr-Sp-Phl	-"-	5.123	5.167	0.087	5.87			5.29	1.20	2.80	0.919	729	1.9	69.9	20.4	0.8	2.7	30	5.39	1.9			
			5.210																					
Obn69-13	Sp Hzb	-"-	5.364	5.364					5.51	0.76	0.33	0.919	923	1.9	74.8	23.8	0.7	0.7	35	5.63	1.9			
Udachnaya, central Siberian Craton, Russia (AR dunites)																								
U220	Megacr-Dunite	Ionov et al. (2010)	5.490	5.490		5.78			5.44	0.68	0.47	0.925	736	2.6	93.4	2.3		4.2	45	5.60	2.6	5.410	5.418	5.580
Uv591-09	Dunite	Ionov et al. (2020a)	5.467	5.418	0.099	6.25			5.48	0.23	1.04	0.922	958	2.6	89.8	5.0	4.4	0.7	45	5.64	2.6			
			5.368																					
Uv499-09	Dunite	-"-	5.227	5.322	0.078	5.81	5.49		5.34	0.56	0.83	0.922	1028	2.6	91.8	3.6	3.5	1.0	45	5.50	2.6			
			5.291			5.97																		
			5.340																					
			5.290																					
			5.460																					
Uv95-03	Megacr-Dunite	-"-	5.350	5.400	0.050				5.40	0.34	0.21	0.929		2.9	98.0			1.7	45	5.56	2.9	5.370	5.346	5.508
			5.450																					
Uv83-13	Megacr-Dunite	-"-	5.381	5.362	0.038				5.35	0.55	0.20	0.931		2.6	97.0			3.0	45	5.51	2.6	5.375	5.404	5.566
			5.343																					
Uv569-13	Megacr-Dunite	-"-	5.420	5.375	0.090	5.97			5.42	0.22	0.25	0.925	1000	2.6	98.0	0.3	0.9	1.2	45	5.58	2.6			
			5.330																					

Uv06-17	Megacr-Dunite	-"-	5.498	5.433	0.086				5.50	0.02	0.13	0.929		2.6	99.6	0.3		0.1	45	5.66	2.6			
			5.355																					
			5.359																					
			5.514																					
Uv591-09	Coarse dunite	-"-	5.342	5.396	0.108	6.25			5.47	0.23	1.04	0.922	958	2.6	90.0	5.0	4.4	0.7	45	5.64	2.6			
			5.450																					
Uv529-10	Coarse dunite	-"-	5.433	5.340	0.980	5.96			5.32	0.66	0.44	0.923	999	2.6	91.0	4.4	0.7	3.9	45	5.49	2.6			
			5.270																					
			5.318																					
Uv86-13	Coarse dunite	-"-	5.343	5.342	0.003				5.36	0.48	0.65	0.924	946	2.6	94.0	1.6	2.5	2.2	45	5.52	2.6			
			5.340																					
Uv250-13	Coarse dunite	-"-	5.468	5.509	0.082	5.82			5.34	1.03	0.28	0.929	1130	2.9	93.0	1.5		5.7	45	5.50	2.9			
			5.550																					
Uv575-13	Coarse dunite	-"-	5.166	5.269	0.125	5.91			5.26	0.82	0.36	0.926	783	2.9	93.0	2.1		4.5	45	5.43	2.9			
			5.260																					
			5.380																					
Uv85-14	Coarse dunite	-"-	5.442	5.301	0.146	6.13			5.38	0.27	0.31	0.930	857	2.9	91.0	8.6		0.6	45	5.54	2.9			
			5.202																					
Average Archean			5.260		*5.377*	*5.928*		*5.44*												*5.591*	*0.106*			
Udachnaya, central Siberian Craton, Russia (PPR harzburgites)																								
Uv76-13	Sp Hzb, op-rich	Ionov et al. (2020a)	5.209	5.209					5.48	1.19	0.42	0.933	903	2	54.6	44.3	0.0	1.2	35	5.61	2	5.215	5.310	5.422
Uv90-03	Sp Hzb, op-rich	Doucet et al. (2012)	5.097	5.127	0.059	5.78			5.43	1.67	1.31	0.930	845	2	51.9	42.6	4.9	0.6	35	5.56	2			
			5.156			5.70																		
Uv101-3	Sp Hzb, cp	Doucet et al. (2012)	5.200	5.200					5.34	1.56	0.53	0.929	851	2	76.4	20.2	1.9	1.5	30	5.44	2			
																			30					
Uv105-03	Sp Hzb, no cp	-"-	5.415	5.393	0.045	6.48			5.52	0.67	0.33	0.928	881	2	86.4	12.8	0.0	0.8	35	5.65	2			
			5.370																					
Uv107-03	Sp Hzb	-"-	5.090	5.090		6.15			5.22	0.55	0.54	0.924	939	2	86.2	11.1	2.0	0.7	35	5.35	2			
Uv101-11	Sp Hzb, op-rich	Ionov et al. (2020a)	5.293	5.344	0.102		5.62		5.48	1.89	0.91	0.929	961	2	51.7	46.0	1.9		30	5.60	2			
			5.395																					
Uv402-09	Sp Hzb		5.356	5.315	0.083	5.69			5.35	0.13	0.86	0.923	762	2	89.3	10.3	0.0	0.4	35	5.47	2			
			5.273																					
Uv419 -09	Gr Hzb	Doucet et al. (2013)	5.248	5.248					5.35	1.00	1.37	0.922	931	2	69.4	21.5	4.5	4.6	30	5.45	2			
Uv454-09	Sp Hzb	Doucet et al. (2012)	5.282	5.314	0.063	6.16			5.50	1.22	0.47	0.926	818	2	75.2	22.2	1.6	1.0	30	5.61	2			
			5.345			5.93																		
Uv504-09	Sp Hzb, cp	-"-	5.480	5.415	0.130				5.51	0.16	0.82	0.919	839	2	86.9	9.7	3.3	0.1	30	5.62	2			
			5.350																					
Uv542-09	Sp Hzb	Ionov et al. (2020a)	5.360	5.339	0.043				5.44	0.90	0.30	0.934	842	2	82.9	16.3		0.8	35	5.57	2			
			5.317																					
Uv-573-09	Transit. Gr Lh	Doucet et al. (2013)	5.260	5.260					5.24	1.59	1.70	0.911	1283	2	69.6	17.4	5.6	7.4	30	5.35	2			
Uv585-09	Sp Hzb, op-rich	Doucet et al. (2012)	5.454	5.263	0.128	5.68			5.41	1.19	0.82	0.924	832	2	61.4	35.0	3.0	0.6	30	5.52	2			
			5.224			5.63																		
			5.185																					
			5.190																					
Uv590-09	Sp Hzb	Doucet et al. (2012)	5.070	5.070					5.17	0.40	0.21	0.924	830	2	83.9	15.4	0.0	0.7	35	5.30	2			
Uv600-09	Sp Hzb, cp	Doucet et al. (2012)	5.340	5.335	0.010				5.41	0.57	0.63	0.927	912	2	86.2	10.5	2.5	0.8	35	5.53	2			
			5.330							0.57		0.927							35					
Uv604-09	Gr Hzb	Doucet et al. (2013)	5.023	5.023					5.17	0.92	0.90	0.923	939	2	84.6	10.7	0.0	4.7	35	5.21	2			
KC137-08	Sp Hzb	-"-	5.432	5.260	0.1748	6.46			5.33	9.00	0.26	0.925	771	2	93.3	6.3	0.0	0.4	35	5.46	2			
			5.159			6.44																		
			5.19																					
U4	Transit. Gr Lh	Ionov et al. (2010)	5.140	5.140		5.56			5.18	0.95	1.45	0.913	1286	2	70.8	19.3	5.5	4.4	30	5.29	2			
U15	Sp Hzb	-"-	5.050	5.060	0.020	5.77			5.30	1.34	1.03	0.930	760	2	63.3	30.7	4.1	0.4	30	5.41	2			

			5.070																				
U24	Sp Hzb	-"-	5.150	5.150		5.93		5.27	0.88	0.63	0.928	885	2	83.3	13.6	2.4	0.7	30	5.37	2			
U29	Gar Lh	-"-	5.020	5.020				5.17	1.13	1.43	0.921	921	2	68.6	20.2	5.6	2.1	30	5.27	2			
U52	Sp Hzb	-"-	5.100	5.100				5.34	1.40	0.99	0.930	894	2	61.9	34.0	3.7	0.4	30	5.45	2			
U64	Gr Hzb	-"-	5.040	5.040		5.25		5.09	0.50	0.94	0.910	1176	2	77.1	16.6	3.8	2.5	35	5.22	2			
U71	Transit. Gr Hzb	-"-	5.150	5.150				5.10	1.50	1.53	0.912	1300	2	76.5	11.2	4.7	7.6	30	5.21	2			
U260	Gar Hzb	-"-	5.388	5.388				5.44	0.27	1.14	0.918	871	2	90.0	4.5	4.5	1.0	35	5.56	2			
U280	Gr Hzb	-"-	5.131	5.131		5.79		5.14	1.00	0.60	0.919	857	2	85.1	9.3	1.3	4.3	30	5.25	2			
U283	Gr Hzb	-"-	5.254	5.254	0.000			5.28	0.91	0.59	0.920	862	2	84.5	10.2	1.4	3.7	30	5.39	2			
			5.254																				
U501	Gr Hzb	-"-	5.140	5.187	0.093			5.17	1.43	1.27	0.913	866	2	82.4	6.6	4.3	6.7	30	5.28	2			
			5.233																				
U503	Sheared Gr Hzb	-"-	5.065	5.065		5.58		5.06	1.12	1.26	0.912	1288	2	75.2	14.0	3.7	7.1	30	5.17	2			
U504	Sp Hzb	-"-	5.348	5.348		5.86		5.42	0.85	0.57	0.929	965	2	84.3	12.7	2.0	0.0	30	5.53	2			
U506	Gr Hzb	-"-	5.130	5.130				5.17	0.91	0.94	0.926	1002	2	77.2	16.0	2.6	4.2	30	5.28	2			
U508	Gr Hzb	-"-	5.378	5.378				5.34	1.42	0.83	0.918	883	2	85.4	7.9		6.7	30	5.45	2			
U1123	Sp Hzb	-"-	5.255	5.255		5.79		5.44	1.57	0.72	0.929	847	2	63.1	33.6	2.6	0.7	30	5.55	2			
U1147	Gr Hzb	-"-	5.312	5.312				5.35	1.78	0.81	0.925	994	2	64.5	26.4	1.0	8.1	30	5.46	2			
U1188	Gr Hzb	-"-	5.220	5.220		5.88		5.24	0.98	1.04	0.917	1340	2	69.9	23.1	2.0	5.0	30	5.34	2			
Tok, SE Siberian Craton, Russia (PPR harzburgites)																							
TOK9510-17	Gr Hzb	Ionov et al. (2006)	5.120	5.195	0.084	5.64	5.41	5.27	1.64	0.92	0.904	1011	1.8	75.6	19.5	3.6	1.3	20	5.34	1.5	5.116	5.195	5.276
			5.264																				
			5.201			5.89	5.38																
TOK9502-9	Gr Hzb	-"-	4.969	5.038	0.137	5.51		5.12	0.75	0.72	0.913	874	1.8	79.2	15.7	3.1	0.5	25	5.21	1.5			
			5.106																				
Vitim, southern Siberia (off-craton Sp peridotite)																							
V314-5	Sp Hzb	Ionov & Hofmann (200	5.387	5.319	0.137			5.44	1.25	0.56	0.889	1033	0.3	75.7	22.0	1.8		25	5.53	0.3	5.319	5.445	5.535
			5.25																				
Tariat, central Mongolia (off-craton Sp peridotites)																							
Mo95	Sp Lh, low-cp	Ionov & Hofmann (200	5.295	5.222	0.125	5.52		5.25	1.54	1.38	0.908		0.3					20	5.32	0.3	5.190	5.265	5.319
			5.100																				
			5.272																				
MHP-1 1.08	Sp Lh, px-rich	-"-	5.195	5.195		5.63		5.27	3.92	3.12	0.892		0.3					5	5.29	0.3			
Mo4500-18	Sp Hzb, cp	-"-	5.020	5.075	0.110	5.56		5.21	0.72	0.52	0.919		0.3					25	5.30	0.3			
			5.130																				
Mo+1 79-4	Sp Lh, low-cp	-"-	5.191	5.191		5.49		5.25	2.90	2.02	0.904		0.3					10	5.28	0.3			
Mo8530-24	Sp Lh, low-cp	-"-	5.267	5.267		5.76	5.45	5.35	1.99	1.13	0.899		0.3					15	5.40	0.3			
Dariganga, SE Mongolia (off-craton Sp peridotites)																							
DAR9419-5	Sp Hzb, cp	Unpublished	5.156	5.105	0.092			5.19	0.84	0.62	0.912		0.3					20	5.26	0.3	5.102	5.177	5.249
			5.145																				
			5.015																				
BN-8	Sp Hzb, cp	-"-	4.920	5.098	0.194	5.51	5.31	5.16	1.26	0.75	0.919		0.3	74.0	22.0	3.0	2.0	20	5.23	0.3			
			5.291																				
			4.942																				
			5.238																				
Far eastern Russia (off-craton Sp peridotites)																							
Sv32	Sp Lh, low-cp	Ionov et al. (2020b)	5.255	5.255		5.54	5.38	5.31	1.52	1.38	0.915	988	1.8	72.0	21.5	6.0	0.9	20	5.38	1.8	5.255	5.311	5.383
Spitsbergen, Svalbard (off-craton Sp peridotites)																							
SB2161-7-3		Unpublished	5.335	5.337	0.003	5.51	5.43	5.34	1.53	0.97	0.915	929	1.8	81.1	13.1	4.0	1.7	20	5.41	1.8	5.332	5.397	5.475
			5.338																				
SB2161-7-2		-"-	5.313	5.263	0.101	5.77		5.35	0.67	0.46	0.917	1018	1.8	81.0	17.1	1.2	0.6	25	5.44	1.8			
			5.212																				
SB2166-5		-"-	5.397	5.397		5.96		5.50	1.63	0.84	0.916	950	1.8	76.0	19.3	3.2	1.4	20	5.58	1.8			

Proterozoic Av					5.220	0.28		5.318											5.425					
Phanerozoic					5.168	5.787		5.289											0.097					
Avacha, Kamchatka arc (CZ)		Ionov (2010), Bénard, Ionov (2013)																						
Av-1	Sp Hzb ±cp,am		5.020	5.020		5.61			5.14	0.50	0.71	0.909	903	0.1	76.9	20.1	2.4	0.6	30	5.25	0.2	5.167	5.304	5.411
Av-2	-"-	-"-	5.384	5.347	0.074	5.75			5.44	0.55	0.89	0.909	906	0.1	74.8	21.1	3.1	0.6	30	5.54	0.2			
			5.310			5.73																		
Av-3	-"-	-"-	4.971	4.996	0.049	5.90	5.45		5.17	0.41	0.83	0.908	946	0.1	76.9	19.5	2.9	0.5	30	5.28	0.2			
			5.020			5.71																		
Av-4	-"-	-"-	5.110	5.090	0.040	5.58			5.24	0.75	0.90	0.906	920	0.1	66.8	29.6	2.8	0.6	28	5.34	0.2			
			5.070			5.74																		
Av-6	-"-	-"-	5.290	5.161	0.134	5.68			5.26	0.48	0.60	0.909	993	0.1	77.3	19.8	1.4	0.6	30	5.37	0.2			
			5.071																					
			5.122																					
Av-7	-"-	-"-	5.260	5.260		5.84			5.37	0.42	0.54	0.909	916	0.1	78.9	18.8	1.7	0.5	30	5.48	0.2			
Av-8	-"-	-"-				5.94				0.78	0.91	0.911	958	0.1	69.2	26.4	2.6	1.1	28		0.2			
Av-9	-"-	-"-				5.67				0.50	0.58	0.909	923	0.1	79.1	18.0	1.9	0.8	30		0.2			
Av-10	-"-	-"-				5.71				0.62	0.87	0.910	949	0.1	73.2	23.3	2.9	0.6	30		0.2			
Av-11	-"-	-"-	5.120	5.120		5.85			5.26	0.44	0.53	0.910	930	0.1	78.3	19.2	1.7	0.7	30	5.37	0.2			
Av-12	-"-	-"-	5.270			5.85			5.38	0.62	0.55	0.909	917	0.1	77.3	19.8	1.5	0.7	30	5.49	0.2			
Av-13	-"-	-"-				6.02				0.53	0.64	0.910	954	0.1	76.8	19.8	1.6	0.7	30		0.2			
Av-14	-"-	-"-				6.00				0.85	0.84	0.909	915	0.1	69.7	26.3	2.6	0.8	28		0.2			
Av-15	-"-	-"-	5.270	5.270		5.92			5.43	0.63	1.28	0.909	960	0.1	73.6	20.9	4.7	0.7	30	5.54	0.2			
Av-16	-"-	-"-	5.240	5.240		5.70			5.33	0.52	0.73	0.916	989	0.1	76.5	20.3	2.2	0.8	30	5.44	0.2			
Av-17	-"-	-"-	5.280			5.93			5.41	0.50	0.55	0.914	895	0.1	79.2	18.6	1.9	0.3	30	5.52	0.2			
W Bismarck arc		Tollan (2014); Tollan et al. (2015)																						
A2	Sp Hzb		5.264	5.232	0.065	5.91			5.37			0.917	930	1	77.2	21.2	1.1	0.6	20	5.45	0.1	5.149	5.278	5.350
			5.199			5.84																		
A3	Sp Hrz, reacted		5.191	5.198	0.015	5.96			5.35			0.918	930	0.3	77.9	20.3	1.1	0.7	20	5.42	0.1			
			5.206			5.97																		
A3 neoblasts	Sp Hrz, reacted		5.212	5.216	0.009				5.34			0.913	930	0.3	77.9	20.3	1.1	0.7	20	5.41	0.1			
			5.221																					
A5	Sp Hrz, reacted		5.141	5.122	0.038	5.77			5.25			0.916	930	1.1	77.9	20.3	1.1	0.7	20	5.32	0.1			
			5.103																					
A5a	Sp Hrz		5.101	5.076	0.049				5.20				930	1.1	77.9	20.3	1.1	0.7	20	5.27	0.1			
			5.052																					
B1	Residual		5.117	5.161	0.047	5.93			5.34			0.918	930	0.3	75.1	22.7	1.5	0.6	20	5.41	0.1			
			5.193			5.91																		
			5.175																					
B3	Sp Hrz, reacted		5.140	5.171	0.032	5.75			5.25			0.917	930	0.7	86.4	12.3	1.1	0.2	20	5.32	0.1			
			5.203																					
			5.173																					
B5	Sp Hrz, reacted		5.185	5.152	0.066	5.83			5.29			0.917	930	0.6	77.9	20.3	1.1	0.7	20	5.36	0.1			
			5.119			5.69																		
						5.89																		
B6	Pyroxenite					5.67							930	0.3					20		0.1			
						5.67																		
D4	Sp-Hrz, Residual		5.191	5.094	0.079	5.85			5.25			0.909	930	0.1	77.9	20.3	1.1	0.7	20	5.32	0.1			
			5.064																					
			5.006																					
			5.117																					
D7	Sp Hrz, reacted		5.139	5.098	0.082	5.74			5.18			0.922	930	0.4	84.8	13.2	1.0	1.0	20	5.25	0.1			
			5.057			5.87																		

E1	Sp HrzResidual				5.97			0.907	930	0.3					20		0.1
					6.01												
E3	Sp Hrz, reacted	5.137	5.123	0.029	5.76		5.25	0.905	930	0.5	77.9	20.3	1.1	0.7	20	5.32	0.1
		5.108			5.91						75.0	25.0					0.1
					5.84						75.0	25.0					
**Whole Dataset for mantle peridotites**		**Olivine**	**Ol av**		**Opx**	**Cpx**	**Bulk**										
**Average**		**5.245**	**5.239**		**5.764**	**5.470**	**5.337**		**926**		**77.9**	**18.1**	**2.7**	**1.9**	**29.9**	**5.448**	
**±1st dev**		**0.134**	**0.125**		**0.609**	**0.124**	**0.129**		**128**		**10.2**	**9.7**	**2.0**	**2.0**	**8.9**	**0.143**	
**number**	**104**	**181**	**97**		**91**	**11**	**97**										

Abbreviations: Hzb, harzburgite; Lh, lherzolite; ol, olivine; op, orthopyroxene; cp, clinopyroxene; gr, garnet; sp, spinel; phl, phlogopite.

Data sources:

Ionov et al. (2006): Ionov D.A., Shirey S.B., Weis D., Brügmann G. (2006). Os-Hf-Sr-Nd isotope and PGE systematics of spinel peridotite xenoliths from Tok, SE Siberian craton: Effects of pervasive metasomatism in shallow refractory mantle. Earth Planet. Sci. Lett. 241, N°1-2, 47-64.

Ionov & Hofmann (2007): Ionov D.A. and Hofmann A.W. (2007). Depth of formation of subcontinental off-craton peridotites. Earth Planet. Sci. Lett. 261, N°3-4, 620-634. DOI: 10.1016/j.epsl.2007.07.036

Ionov et al. (2010): Ionov D.A., Doucet L.S., Ashchepkov I.V. (2010) Composition of the lithospheric mantle in the Siberian craton: New constraints from fresh peridotites in the Udachnaya-East kimberlite. J. Petrology 51 (11): 2177-2210. doi: 10.1093/petrology/egq05.

Doucet et al. (2012): Doucet L.S., Ionov D.A., (2012) Depth, degrees and tectonic settings of mantle melting during craton formation: inferences from major and trace element compositions of spinel harzburgite xenoliths from the Udachnaya kimberlite, central Siberia. EPSL. 359-360, 206-218

Doucet et al. (2013): Doucet L.S., Ionov D.A., Golovin A.V. (2013) The origin of coarse garnet peridotites in cratonic lithosphere: new data on xenoliths from the Udachnaya kimberlite, central Siberia. Contrib. Miner. Petrol. 165 (6), 1225-1242, doi:10.1007/s00410-013-0855-8.

Ionov et al. (2015a): Ionov DA, Doucet LS, wt al (2015) Post-Archean formation of the lithospheric mantle in the central Siberian craton: Re-Os and PGE study of peridotite xenoliths from the Udachnaya kimberlite. Geochim. Cosmochim. Acta 165, 466-483

Ionov et al. (2015b): The age and history of the lithospheric mantle of the Siberian craton: Re-Os and PGE study of peridotite xenoliths from the Obnazhennaya kimberlite. Earth Planet. Sci. Lett. 428, 108-119, doi: 10.1016/j.epsl.2015.07.007.

Ionov et al. (2018): Ionov DA, Doucet LS, Xu Y, et al (2018) Reworking of Archean mantle in the NE Siberian craton by carbonatite and silicate melt metasomatism: evidence from a carbonate-bearing, dunite-to-websterite xenolith suite from the Obnazhennaya kimberlite. G. C. A, 224, 132-153

Carlson & Ionov (2019): Carlson RW, Ionov DA (2019) Compositional characteristics of the MORB mantle and Bulk Silicate Earth based on spinel peridotites from the Tariat region, Mongolia. Geochim. Cosmochim. Acta 257, 206-223, doi: 10.1016/j.gca.2019.05.010

Ionov et al. (2020a): Ionov DA, Liu Z, Li J, Golovin AG, Korsakov AV, Xu Y (2020) The age and origin of cratonic lithospheric mantle: Archean dunites vs. Paleoproterozoic harzburgites from the Udachnaya kimberlite, Siberian craton. Geochim. Cosmochim. Acta, 281, 67-90.

Ionov et al. (2020b): Ionov DA, Guo P, Nelson WR, Shirey SB, Willbold M (2020) Paleoproterozoic melt-depleted lithospheric mantle in the Khanka block, far eastern Russia: inferences for mobile belts bordering the North China and Siberian cratons. Geochim. Cosmochim. Acta 270, 95-111

Kopylova MG, JK Russell, H Cookenboo (1999) Petrology of peridotite and pyroxenite xenoliths from the Jericho kimberlite: implications for the thermal state of the mantle beneath the Slave craton, northern Canada. Journal of Petrology 40 (1), 79-104

Kopylova MG, JK Russell (2000) Chemical stratification of cratonic lithosphere: constraints from the Northern Slave craton, Canada. Earth and Planetary Science Letters 181 (1-2), 71-87

Tollan PME (2014) Tale of Two Arcs: Petrogenesis of Ultramafic Xenoliths Sampling the Upper Mantle Wedge Beneath the West Bismarck Island Arc. Doctoral thesis, Durham University.

Tollan PME, HSC O'Neill, J Hermann, A Benedictus, RJ Arculus.(2015) Frozen melt–rock reaction in a peridotite xenolith from sub-arc mantle recorded by diffusion of trace elements and water in olivine. Earth and Planetary Science Letters 422, 169-181

Bénard A, Ionov DA (2013) Melt- and fluid-rock interaction in supra-subduction lithospheric mantle: Evidence from andesite-hosted veined peridotite xenoliths. J. Petrology 54: 2399-2378

Supplementary Table 2. Triple Oxygen Isotope Analyses of olivine and orthopyroxene in studied mantle nodules

	Age, Ga	$\delta'^{17}O$ ‰	$\delta'^{18}O$ ‰	$\delta^{18}O$ ‰	$\Delta'^{17}O$ ‰	±1stdev	p values
Archean							
Obn22-13 ol-1	2.9	2.753	5.2996	5.420	-0.059	0.007	
Obn1017 ol-3	2.9	2.710	5.2031	5.350	-0.050	0.004	
Obn1017 ol-3	2.9	2.781	5.3337	5.350	-0.049	0.006	
Obn37-13 ol	2.9	2.803	5.3562	5.410	-0.039	0.009	
Uv499-09 ol	2.6	2.729	5.2099	5.260	-0.035	0.015	
Uv591-09 ol	2.6	2.793	5.3432	5.420	-0.041	0.010	
JF15-09 ol-1	3.0	2.701	5.1538	5.260	-0.034	0.010	
JF15-01 ol-1	3.0	2.733	5.2467	5.250	-0.051	0.009	
JF15-01 ol-1	3.0	2.858	5.4833	5.250	-0.051	0.009	AR vs PR
				Average	-0.045	0.009	0.1, fail
Proterozoic							
LGS26 ol	2.1	2.720	5.2100	5.280	-0.044	0.013	
LGS22 M1 ol-1	2.1	2.692	5.1630	5.110	-0.047	0.014	
LGS22-M5 ol-1	2.1	2.901	5.5886	5.210	-0.064	0.014	
Obn01-13 ol-1	1.9	2.846	5.4844	5.350	-0.064	0.006	
Obn08-13 ol-1	1.9	2.749	5.2992	5.170	-0.062	0.010	
Obn69-13 ol-1	1.9	2.944	5.6616	5.360	-0.060	0.009	
UV105-03 ol-1	2	2.714	5.1937	5.420	-0.041	0.011	
UV419-09 ol-1	2	2.731	5.2162	5.250	-0.036	0.012	
UV504-09 ol-1	2	2.736	5.2469	5.340	-0.047	0.014	
UV504-09 ol-1	2	2.667	5.1011	5.340	-0.039	0.007	
SB2161-7-2 ol-1	1.8	2.837	5.4449	5.260	-0.052	0.011	
TOK9510-17 ol	1.8	2.727	5.2370	5.190	-0.051	0.005	PR vs Phan
				Average	-0.051	0.010	0.054, fail
Phanerozoic							
AV20 opx vein	0.1	3.044	5.8348	5.852	-0.052	0.005	
AV19+13+20 opx vein	0.1	3.009	5.7791	5.796	-0.057	0.003	
AV-21 opx vein	0.1	2.756	5.2921	5.306	-0.052	0.014	
AV1 opx vein	0.1	2.450	4.7225	5.610	-0.055	0.005	
AV-9 opx vein	0.1	2.556	4.9035	5.670	-0.045	0.007	AR vs post-AR
				Average	-0.052	0.005	0.36, fail

Ol-1 means a single crystal

primed notation use linearization: $\delta'^{1x}O=1000\ \ln(\delta^{1x}O/1000+1)$, where x is 18 or 17

T-test (P-value) does not differentiate Δ'17O ‰ values of different ages

Supplementary Table 3 Data from Mattey et al (1994) with assigned ages. Age 1 refers to the dominant age of the host lithosphere. Age 2 assumes recent reworking of Western N American lithosphere

Locality, lithology	Sample #	$\delta^{18}O$, ‰ VSMOW			Age Model #1	Age Model #2
		Ol	Opx	Cpx	Ga	Ga
Geronimo 20 sp-lhz	ZH1	4.93	5.61	5.28	1.7	0.1
Geronimo 2° sp-lhz	1A 20-2	5.27	5.79	5.54	1.7	0.1
Geronimo 2° sp-lhz	1A 20-7	5.17	5.75	5.45	1.7	0.1
Geronimo 2° sp-lhz	lA 20-9WR	5.42	5.81	5.72	1.7	0.1
Geronimo 2° sp-lhz	IA 20-9V	5.20	5.86	5.57	1.7	0.1
Dish Hill 2° sp-lhz	1A h ENO2	4.94	5.77	5.25	0.05	0.1
Dish Hill 2° sp-lhz	1A h Ba-2-1	5.14	5.4	5.62	0.05	0.1
Geronimo 2° sp-lhz	1B 20-12	5.03	5.49	5.41	1.7	0.1
Geronimo 2° sp-lhz	1B 21-6	5.15	5.58	5.36	1.7	0.1
Geronimo 2° sp-lhz	1B ZH5	5.18	5.72	5.63	1.7	0.1
Geronimo 20 sp-lhz	IB h 22-16A	5.15	5.70	5.56	1.7	0.1
Dish Hill 20 sp-lhz	I B h ENO1	5.05	5.52	5.38	0.05	0.1
Dish Hill 2° sp-lhz	IB h Ba-l-72	5.12	5.76	5.69	0.05	0.1
Geronimo 2° sp-lhz	2 20-6	5.08	5.33		1.7	0.1
Geronimo 2° sp-lhz	2 20-8	4.97	5.44		1.7	0.1
Massif Central sp-lhz	1A 91-3	5.24	5.89	5.84	0.35	
Massif Central sp-lhz	1A 91-4	5.28	5.86	5.90	0.35	
Massif Central sp-lhz	1A 91-8	5.25	5.89	5.65	0.35	
Massif Central sp-lhz	IA 91-9	5.15	5.66	5.68	0.35	
Massif Central sp-lhz	1B 83-67	5.00	5.73	5.51	0.35	
Thumb 21 gt-lhz	h N077	5.41	5.73	5.40	1.8	
Thumb 21 gt-lhz	h H077	5.47	5.75	5.53	1.8	
Thumb 21 gt-lhz	h A082	5.24	5.53	5.64	1.8	
Thumb 21 gt-lhz	126	5.47	5.68	5.48	1.8	
Thumb 2j gt-lhz	I078	5.50	5.68	5.50	1.8	
Thumb 21 gt-lhz	R077	5.27	5.45	5.44	1.8	
Thumb 21 gt-lhz	h D076	5.26	5.56	5.42	1.8	
Thumb 21 gt-lhz	145	5.22	5.70	5.58	1.8	
Thumb 2j gt-lhz	U078	5.05	5.73	5.58	1.8	
Thumb 21 gt-lhz	B082	5.19	5.56	5.49	1.8	
Thumb 21 gt-lhz	160	5.25	5.48	5.32	1.8	
Thumb 2L gt-lhz	h 140	5.18	5.70	5.51	1.8	
Udachnaya 22 mega-dun	Lo-T D UV69/76	5.25			2.6	
Udachnaya 22	UV255/75	5.22	5.55		2.6	
Udachnaya 22 mega-dun Lo-T	UV47/76	5.19			2.6	
Udachnaya 22 mega-dun Lo-T	UV49/76	5.12			2.6	
Udachnaya 22 mega-dun Lo-T	UV70/76	5.25			2.6	
Udachnaya 22 gt-lhz Lo-T	UV 413189	5.28	5.90		2.6	
Udachnaya 22 gt-lhz Lo-T	UV417/89	5.11	5.84		2.6	
Udachnaya 22 gt-lhz Hi-T	UV61/91	5.25	5.57	5.59	2.6	
Premier 6 gt-lhz Hi-T	PHN 5239	5.36	5.36		3	
Premier 6 gt-lhz Hi-T	PHN 5267	5.28	5.63	5.62	3	
Premier 6 gt-lhz Hi-T	PHN 5246	5.29	5.78	5.71	3	
Premier 6 gt-lhz Hi-T	FRB 909	5.15	5.47	5.54	3	
Premier 6 sp-lhz Lo-T	PHN 5273	5.31	5.70	5.85	3	
Premier 6 sp-lhz Lo-T	PHN 5275	5.19	5.84	5.59	3	
Premier 6 sp/gt-lhz Lo-T	FRB 1350	5.3	5.81	5.76	3	
Jagersfontein gt-hz	JFL HI	5.07	5.84	5.56	2.7	

Jagersfontein gt-lhz Hi-T	JFL L1	5.23	5.66	5.66	2.7	
Bultfontein 25 PKP	h AJE 125	5.09	5.87		2.7	
Bultfontein 25 PGP	h AJE 159	5.11	5.78	5.51	2.7	
Bultfontein 25 GP	h AJE 164	5.15	5.64	5.44	2.7	
Bultfontein 25 PKP	h AJE 168	5.18	6.10	5.75	2.7	
Kaalvallei perid	D KV9	5.13			2.5	
Finsch 22,23 gt-lhz	D F 556	5.15			2.5	
Finsch 22,23 gt-hz	D F 865	5.07	5.63		2.5	
Finsch 22,23 gt-hz	D F 866	5.08	5.76		2.5	
Finsch 22,23 gt-lhz	JJG 147	5.15	5.63	5.62	2.5	
Finsch SDI	hz 13A (1) §				2.5	
Finsch SDI	P 8(6)				2.5	
Finsch SDI	8(4)				2.5	
Finsch SDI	8(7)				2.5	
Finsch SDI	8(3)				2.5	
Finsch SDI	8(2)				2.5	
Baja sp	CSQ32e	5.05			0.1	0.1
Dish Hill s sp-lhz	33b-18	4.99	5.62	5.42	0.05	
Dish Hill s sp-lhz	DH-AA	5.05	5.39	5.60	0.05	
Dish Hill 8 sp-lhz	DH-A	4.95	5.55	5.41	0.05	
Dish Hill 8 sp-lhz	DH-B	5.28	5.86	5.57	0.05	
Dish Hill 8 sp-lhz	DH-L	4.88	5.74	5.80	0.05	
Hawaii, gt-lhz SL	C-18	5.21	5.66	5.83	0.1	
Hawaii	CLC20	5.29	5.75	5.68	0.1	
	NB40	5.07			0.1	
	BN55	5.09			0.1	
	LS	5.18			0.1	
	MB19	4.96	5.5	5.49	0.1	

Data from Chazot et al. (1997)

Bir Ali Yemen Gulf af Aden Spinel Lherzolites

	$\delta^{18}O$, other mineral					
BA5		5.20	5.85	5.70		0.1
BA7	4.75 Sp	5.12	5.9	5.60		0.1
BA8	4.54 Sp	5.22	5.84	5.53		0.1
Ataq						
JK1		5.20	5.68	5.71		0.1
JK4	4.00 Sp	5.34	5.93	5.74		0.1
JK5		5.28	5.93	5.77		0.1
Ataq hydrous						
JK2	5.35 Amph	5.21	5.72	5.69		0.1
JK2 vein	5.45 Amph	5.34	5.82	5.63		0.1
JK3	5.53 Amph, 3.99 Sp	5.21	5.90	5.72		0.1
JK7	5.35 Amph	5.37	5.83	5.77		0.1
JK8	5.37 Amph	5.28	5.98	5.53		0.1
Nunivak W coast of Alaska small Island in the Bering Sea						
UM1	5.34 Amph 3.84 Sp	5.29	5.92	5.62		0.1
10051	5.54 Phlog	5.35	5.66	5.78		0.1

Sp -spinel, Amph -amphibole, Phlog - phlogopite, gt- gartet
hz-harzburgite, lhz lherzolite, dun- dunite, mega- mehacrystic

Supplementary Table 4 Comparison of average δ18O ‰ values of mantle peridotites in this dataset with that of Mattey et al. (1994)

	this study $\delta^{18}O$ ‰	n	Mattey et al. (1994) $\delta^{18}O$ ‰	n
Olivine	**5.239±0.124**	**97**	5.18±0.14	76
Orthopyroxene	**5.821±0.212**	**91**	5.69±0.14	54
Clinopyroxene	**5.47±0.124**	**11**	5.57±0.16	57
Bulk calculated	**5.337±0.128**	**97**	5.337±0.112	57

Supplementary Table 5. T Test of variably split dataset and Line Fit Statistics

t tests of our dataset	$\delta^{18}O$ ‰	n
Archean:		
(craton)		
Olivine	**5.377±0.073**	**20**
Orthopyroxene	**5.928±0.185**	**13**
Bulk	**5.439±0.115**	**20**
Cont Lithospheric Mantle (CLM	**5.591±0.106**	**20**
(melting degree corrected)		
Proterozoic:		
(off-craton)		
Olivine	**5.220±0.117**	**47**
Orthopyroxene	**5.824±0.287**	**32**
Bulk	**5.318±0.132**	**47**
Cont Lithospheric Mantle	**5.425±0.138**	**47**
(melting degree co		
Phanerozoic:		
-craton and island arcs)		
Olivine	**5.168±0.089**	**27**
Orthopyroxene	**5.787±0.145**	**31**
Bulk	**5.289±0.088**	**27**
Cont Lithospheric Mantle	**5.373±0.097**	**27**
(melting degree corrected)		

t- test, statistical significance of difference
p value <0.05 (95% conf) =pass

Archean vs Proterozoic		p-values	result
	Ol	2.3E-06	pass
	Bulk	1.1E-03	pass
	CLM	2.2E-05	pass
Proterozoic vs Phanerozoic			
	Ol	1.7E-04	pass
	Bulk	9.0E-02	no
	Bulk Corr	1.5E-02	pass
Archean vs post-Archean			
	Ol	1.6E-11	pass
	Bulk	2.0E-05	pass
	Bulk Corr	3.0E-08	pass

LINEAR FIT STATISTICS

All Data, using averages for each mantle nodule, n=104

	Olivine	Bulk	Bulk corrected for melting
slope	0.067	0.048	0.070
±slope	0.012	0.013	0.014
intercept	5.128	5.260	5.330
±intercept	0.023	0.026	0.027
r2	0.253	0.120	0.210
Result	TRUE	TRUE	TRUE
Mantle at 4Ga:	5.396‰		5.61‰

p values

T TEST*	Olivine	Bulk	Bulk corrected for melting
Ar vs post_AR	4.45E-10	1.68E-05	3.13E-08
	Pass	Pass	Pass
AR vs PR	1.39E-07	4.93E-04	6.90E-06
	Pass	Pass	Pass
<2 Ga vs >2 Ga	5.78E-04	2.01E-02	4.27E-04
	Pass	Pass	Pass
PR vs Phan	2.30E-02	7.60E-02	1.60E-02
	Pass	Pass	Pass

Al Data Using averages for locality, n=15

	Olivine	Bulk	Bulk corrected for melting
slope	0.065	0.059	0.083
±slope	0.017	0.023	0.025
intercept	5.150	5.264	5.331
±intercept	0.033	0.043	0.047
r2	0.523	0.342	0.462
Result	TRUE	TRUE	TRUE

p values

T TEST	Ol	Bulk	Bulk corrected for melting
AR vs post AR	0.00048	0.01675	0.00348
	Pass	Pass	Pass
AR vs PR	0.00584	0.05774	0.01629
	Pass	No	Pass
>2 Ga vs <2 Ga	0.01147	0.04499	0.00970
	Pass	Pass	Pass
PR vs Phan	0.09312	0.15691	0.12098
	No	No	No

*1 tailed, Pass criteria p value <0.05
